# Characterization of Polyisobutylene Succinic Anhydride (PIBSA) and Its PIBSI Products from the Reaction of PIBSA with Hexamethylene Diamine

**DOI:** 10.3390/polym15102350

**Published:** 2023-05-17

**Authors:** Franklin Frasca, Jean Duhamel

**Affiliations:** Department of Chemistry, Institute for Polymer Research, Waterloo Institute for Nanotechnology, University of Waterloo, 200 University Avenue West, Waterloo, ON N2L 3G1, Canada; fofrasca@uwaterloo.ca

**Keywords:** gel permeation chromatography, PIBSA, PIBSI dispersants, sums of Gaussians

## Abstract

The nature of the end-groups of a PIBSA sample, namely a polyisobutylene (PIB) sample, where each chain is supposedly terminated at one end with a single succinic anhydride group, was characterized through a combination of pyrene excimer fluorescence (PEF), gel permeation chromatography, and simulations. The PIBSA sample was reacted with different molar ratios of hexamethylene diamine to generate PIBSI molecules with succinimide (SI) groups in the corresponding reaction mixtures. The molecular weight distribution (MWD) of the different reaction mixtures was determined by fitting the gel permeation chromatography traces with sums of Gaussians. Comparison of the experimental MWD of the reaction mixtures with those simulated by assuming that the reaction between succinic anhydride and amine occurs through stochastic encounters led to the conclusion that 36 wt% of the PIBSA sample constituted unmaleated PIB chains. Based on this analysis, the PIBSA sample was found to be constituted of 0.50, 0.38, and 0.12 molar fractions of PIB chains that were singly maleated, unmaleated, and doubly maleated, respectively.

## 1. Introduction

Living polymerization techniques have enabled the preparation of monodisperse polymers that can be modified at one chain end with specific functionalities [1,2,3,4,5] allowing their incorporation into much larger, well-defined macromolecular architectures through a *grafting onto* scheme [6,7,8]. Such reaction schemes enable the synthesis of highly branched macromolecules with controlled architectures, such as arborescent or dendritic polymers [9,10,11,12,13,14] or polymeric bottle brushes [15,16,17]. The *click* reaction between an end-modified polymer (EMP) and the appropriate substrate requires that all linear chains be end-modified with a single functionality [1,2,3,4,5,6,7,8,9,10,11,12,13,14,15,16,17,18]. While such conditions can be routinely achieved in a research laboratory, they can be more challenging in industry settings, where side reactions can result in incomplete end-modification and/or incorporation of more than one functionality per chain. Since control over the structural integrity of the final macromolecule depends on the chemical purity of the EMP to be grafted onto the substrate, defects in the EMP must be carefully identified so that they can be accounted for during the *grafting onto* reaction, particularly when the pure EMP cannot be easily isolated for use in a multistep industrial process.

Unfortunately, the characterization of the functionality of EMPs prepared through an industrial process is often challenging, as highlighted in a recent publication [19]. First, the end groups of a linear chain are present in low abundance, complicating their detection. Second, techniques based on FTIR, UV-Vis, or NMR spectroscopy might yield the average content of a given functionality per unit mass of the EMP but cannot readily assess the absence of functionalization or the single or multiple modifications of a chain end [20]. Third, while relating the number of chain ends to the degree of functionality of an EMP could provide some information about the nature of the modification (absent, single, or multiple), it would require the determination of the number average molecular weight (*M*_n_) of the polymer [20]. Unfortunately, gel permeation chromatography (GPC) is ill-suited to determine the absolute *M*_n_ for shorter polymers that yield little signal with a light scattering or viscosity detector and for which standards with a narrow molecular weight distribution (MWD) might not be readily available to calibrate the instrument [21]. Similarly, the determination of *M*_n_ by mass spectrometry (MS) is more challenging for polydisperse and apolar polymers [22].

**Scheme 1:**
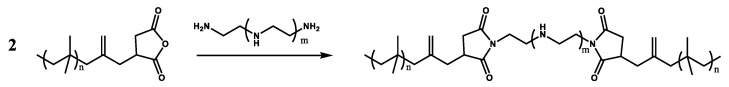
General scheme for the synthesis of *b*-PIBSI dispersants.

These complications affect the characterization of numerous EMPs prepared industrially, including that of polyisobutylene terminated at one end with succinic anhydride (PIBSA). PIBSA can be obtained through the modification of PIB with maleic anhydride via an Alder-ene reaction [23,24]. PIBSA is an important building block for the synthesis of ashless PIBSI dispersants produced by reacting the terminal succinic anhydrides (SA) of two PIBSA molecules with the two terminal primary amines of a linear polyamine-like tetraethylene pentamine (TEPA) or pentaethylene hexamine (PEHA) [25,26,27]. This reaction produces a PIBSI dispersant by generating two succinimides (SI) connecting two PIB chains to the polyamine core (Scheme 1). PIBSI dispersants are added to engine oil to stabilize the polar particles generated in the oil during the normal operation of the engine, which would otherwise agglomerate and lead to oil thickening [25]. The polar polyamine core of a PIBSI dispersant adheres to the polar surface of insoluble particulates in the oil, while the large apolar PIB segments prevent further aggregation, mainly through steric stabilization [27,28].

As discussed in a recent publication [19], the importance of PIBSI dispersants as oil additives requires that their synthesis be carefully controlled through the use of well-characterized PIBSA blocks. Unfortunately, most techniques typically available in polymer science to characterize macromolecules are ineffective in characterizing the functionality of many EMPs, such as PIBSA. While a combination of pyrene excimer fluorescence, UV-Vis absorption, and ^1^H NMR analysis can provide the SA content (*λ*_SA_) and molar fraction (*f*_doubly_) of doubly maleated PIBSA chains (PIBSA_2_) of a PIBSA sample, which had been determined in an earlier study [19] to equal 0.418 mmol/g and 0.19, respectively, the weight fraction (*w_PIB_*) of unmaleated PIB chains remains unknown.

To address this issue, a general methodology is proposed in the present study to determine the weight fraction of unmodified polymers in an EMP sample by using GPC analysis to monitor the reactants and products for a reaction between an EMP sample and a bifunctional linker selected to react effectively with the end groups of the EMP. By taking advantage of the ability of GPC to separate polymer chains according to their size [29,30], GPC analysis of reaction mixtures obtained with different molar equivalents (meq) of EMPs and bifunctional linkers can uncover the amount of unmodified polymer that does not partake in the coupling reaction by taking advantage of the size difference between the doubly and singly appended linkers. The detection of unreacted EMPs in a coupling reaction involving two meq of EMPs and one meq of a bifunctional linker, which should result in the complete consumption of the EMP, is a clear indication that some unmodified polymer is present in the EMP sample, whose weight fraction can be quantified through GPC analysis.

As a case in point, this methodology was applied to a series of reactions referred to as PIBSA–H(*X*) between a PIBSA sample and a bifunctional linker, namely hexamethylene diamine (HMDA), with different amine-to-SA (*X* = *N*_Am_/*N*_SA_) ratios ranging from 0.19 to 1.51. The constituents of the reaction mixtures and their relative contents were characterized by gel permeation chromatography (GPC) analysis, which indicated that a constant amount of PIBSA reactant was unable to react for all amine-to-SA ratios. The unreactive PIBSA species was most likely unmaleated PIB that was unable to participate in the succinimide formation. Careful analysis of the GPC data combined with simulations used to predict the composition of the reaction mixtures led to a highly detailed description of the types of end groups found in the PIBSA sample, with 50, 38, and 12 mol% of the PIB chains being singly maleated, unmaleated, and doubly maleated, respectively. Such detailed end-group analysis of an EMP is required to predict the products of its reaction with a given substrate, and while it was applied to PIBSA as a specific example, the proposed methodology can be extended to any EMP and should thus be of interest to scientists interested in generating complex macromolecular architectures involving reactions with EMPs.

## 2. Materials and Methods

*Chemicals.* Xylene (98.5%, Caledon Laboratory Chemicals), tetrahydrofuran (THF, 99%, Sigma-Aldrich, St. Louis, MO, USA or distilled in glass, 99.9%, Fisher Chemical, Hampton, NH, USA), acetone (HPLC grade, Sigma-Aldrich), and hexamethylene diamine (HMDA, 98%, Sigma-Aldrich) were used without further purification. The PIBSA sample was supplied by Afton Chemical.

*Fourier-transform infrared (FTIR).* A Bruker Tensor 27 FTIR spectrometer was used to obtain the FTIR spectra of the polymer samples. Polymer films were prepared to acquire the FTIR spectra by depositing solutions of the polymer samples in CHCl_3_ onto a NaCl FTIR cell and evaporating the solvent under N_2_. The absorbance was then measured from 1000 to 2000 cm^−1^ and kept around 1.0 to optimize the signal-to-noise ratio.

*Gel permeation chromatography (GPC).* GPC analysis was conducted with a Viscotek GPC max VE 2001 instrument equipped with two PolyAnalytik columns (PolyAnalytik styrene-divinylbenzene SupeRes^TM^ PAS-101 and PAS-103 with exclusion limits of 1.5 × 10^3^ and 7 × 10^4^ Da, respectively, 8 mm × 30 cm) and a triple-detector array, which included a differential refractive index (DRI), viscosity, and static light scattering detector. THF was used as the solvent. The polymer samples were dissolved in the THF from the GPC reservoir to prepare 1 mg/mL polymer solutions. The polymer solutions were filtered through 0.22 μm PTFE filters before injection.

*Fitting the GPC traces with sums of Gaussians*: Since sums of Gaussians were used to fit GPC traces [31,32], the DRI traces were baseline corrected and could be fitted with sums of Gaussians as a function of the variable *x*, which was either the elution volume (*V_el_*) or the degree of polymerization (*X_n_*). The analysis was conducted with the program *x*gauss written in-house, where *x* equals *mono, bi, tri, tetra, penta, or hexa* to fit the DRI traces with a sum of 1–6 Gaussians according to Equation (1), respectively. The sum of Gaussians analysis enabled the mathematical parametrization of the experimental GPC traces, which provided a means to describe them with a small number of parameters. This sum of Gaussians analysis yielded the scaling factor *A* and the parameters *a_i_*, *μ_i_*, and *σ_i_*, representing the normalized pre-Gaussian factor, the average, and the standard deviation of the *i*th Gaussian used to fit the GPC trace. The *x*gaussSNP program series (where *x* = *mono, bi,* and *tri*) was used to fit a DRI trace with Equation (2), divided into two sums of Gaussians. The parameters *a_i_*, *μ_i_*, and *σ_i_* used in the first sum had been pre-determined from the *x*gauss analysis of a DRI trace and were fixed in the analysis with *x*gaussSNP. The parameters *a_j_*, *μ_j_*, and *σ_j_* used in the second sum of Gaussians were optimized in the analysis, along with the scaling factors *A* and *B*.

(1) $$DRI(x)\;=\;A\times \sum_{i=1}^{n}\frac{a_{i}}{\sigma_{i}\sqrt{2\pi }}\exp\left(-\frac{\left(x-\mu_{i}\right)}{2\sigma_{i}^{2}}\right)$$

(2) $$DRI(x)\;=\;A\times \sum_{i=1}^{n}\frac{a_{i}}{\sigma_{i}\sqrt{2\pi }}\exp\left(-\frac{\left(x-\mu_{i}\right)}{2\sigma_{i}^{2}}\right)\;+\;B\times \sum_{j=1}^{m}\frac{a_{j}}{\sigma_{j}\sqrt{2\pi }}\exp\left(-\frac{\left(x-\mu_{j}\right)}{2\sigma_{j}^{2}}\right)$$

In Equations (1) and (2), all pre-Gaussian and scaling factors were forced to remain positive in the analysis. All floating parameters were optimized according to the Levenberg–Marquardt algorithm for both the *x*gauss and *x*gaussSNP fitting programs [33].

*Simulating the composition of a reaction mixture between PIBSA and HMDA.* The programs *reaction3* and *reaction4* were created to simulate the molar fractions of the PIBSA reactants and PIBSI products obtained for a coupling reaction between a given amount of PIBSA and HMDA. The parameters to be input into the programs were the number of molecules to be simulated, the molar fraction *f*_doubly_ of doubly maleated PIB chains in the PIBSA sample, and the ratio *N*_Am_/*N*_SA_ of the number of amines over the number of succinic anhydrides used for a given coupling reaction. These parameters were then employed to create a list of molecules comprised singly maleated PIBSA_1_, doubly maleated PIBSA_2_, and diamine species, with the ratio *N*_PIBSA2_/(*N*_PIBSA1_ + *N*_PIBSA2_) of the number of PIBSA_2_ molecules over the total number of PIBSA molecules given by *f*_doubly_ and the ratio *N*_Am_/(*N*_PIBSA1_ + 2 × *N*_PIBSA2_) being equal to the *N*_Am_/*N*_SA_ ratio. Each molecule in the reaction mixture was defined by its number of PIB chains (*n*_PIB_), succinic anhydrides (*n*_SA_), and amines (*n*_Am_). For instance, a PIBSA_2_ molecule would be referred to as *n*_PIB_ = 1, *n*_SA_ = 2, and *n*_Am_ = 0. Two molecules defined by the parameters *n*_PIB_, *n*_SA_, and *n*_Am_, namely *mol*_1_(*n*_PIB,1_, *n*_SA,1_, *n*_Am,1_) and *mol*_2_(*n*_PIB,2_, *n*_SA,2_, *n*_Am,2_) in the list, were then selected at random from the reaction mixture. If *n*_Am_ ≥ 1 for one molecule and *n*_SA_ ≥ 1 for the other molecule, the two were combined to form a new molecule *mol*_3_(*n*_PIB,3_, *n*_SA,3_, *n*_Am,3_) with *n*_PIB,3_ = *n*_PIB,1_ + *n*_PIB,2_, *n*_SA,3_ = *n*_SA,1_ + *n*_SA,2_ − 1, and *n*_Am,3_ = *n*_Am,1_ + *n*_Am,2_ − 1. The new molecule *mol*_3_ was added to the reaction mixture, and *mol*_1_ and *mol*_2_ were removed before repeating this process until all available amines and/or succinic groups were consumed, i.e., when either *N*_SA_ = 0 or *N*_Am_ = 0 in the reaction mixture.

Two programs were implemented based on this concept. The program *reaction3* allowed for the coupling of any amine and PIBSA molecule with no limit to the oligomerization of PIBSA molecules through the PIBSA_2_ species into *tri*-, *tetra*-, *penta*-PIBSIs, etc. The *reaction4* program limited the coupling products to contain a maximum of 2 PIBs (*n*_PIB_ ≤ 2), assuming that steric hindrance prevented oligomerization into higher-order PIBSIs, essentially limiting the PIBSI products to *m*- and *b*-PIBSI species. The programs output the number of *m*-, *b*-, *tri*-, *tetra*-, *penta*-, *hexa*-, *hepta*-, *octa*-, and *nona*-PIBSIs resulting from the coupling reaction of PIBSA and diamines, which could be used to determine the molar fraction of each PIBSI species in the reaction mixture.

*Calibration of the GPC instrument with polydisperse PIB standards.* The 2nd order polynomial [*Ln*(*M*) = *b*_0_ + *b*_1_*V_el_* + *b*_2_*V_el_*^2^] obtained as Equation (S13) in Supplementary Material was used as the *Ln*(*M*)-*vs*-*V_el_* calibration curve, which was established using the baseline-corrected DRI traces of a set of 3 PIB standards with a broad MWD purchased from Polymer Source. The probability *P*(*M*) of having a molar mass *M* for a PIBSA–H(*X*) mixture was obtained by applying Equation (3), where the function *DRI*(*V_el_*) corresponds to the GPC trace obtained for the PIBSA–H(*X*) mixture with the DRI detector. The derivation of *P*(*M*) is provided in Supplementary Material, where it is given as Equation (S9) [34].

(3) $$P(M)\;=\;\frac{1}{\int_{0}^{\infty }DRI(V_{el})\exp(-\sum_{i=0}^{n}b_{i}V_{el}^{i})dV_{el}}\;\times \frac{DRI(V_{el})}{\exp\left(2\times \sum_{i=0}^{n}b_{i}V_{el}^{i}\right)\times \sum_{i=1}^{n}ib_{i}V_{el}^{i-1}}$$

**Scheme 2:**
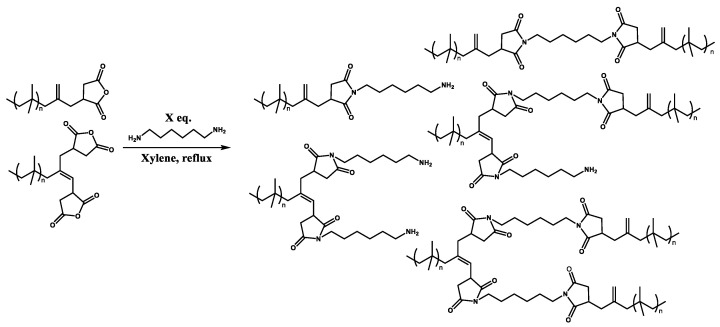
Synthesis of PIBSI–HMDA products.

*Synthesis of PIBSA–HMDA samples.* The PIBSA sample received from Afton Chemical had an *f*_doubly_ of 0.19, as previously determined [19], and was reacted with varying amounts of HMDA to form *m*-PIBSI, *b*-PIBSI, and possibly other higher-order PIBSI products enabled by the presence of PIBSA_2_, according to Scheme 2.

The *N*_Am_/*N*_SA_ ratio was varied from 0.19 to 1.51 in 0.19 increments to study how this ratio would affect the proportion of reactants and products generated during the coupling reactions. The proportion of PIBSA was expected to decrease as the *N*_Am_/*N*_SA_ ratio increased from 0.19 to 1.0, as more amine is provided for coupling PIBSA into *b*-PIBSI and higher-order PIBSI dispersants [33]. The proportion of *m*-PIBSI should increase when the *N*_Am_/*N*_SA_ ratio increases from 1.0 to 1.51, as PIBSA molecules are ‘capped’ by the excess amines, resulting in more *m*-PIBSIs bearing a free amine. The reaction with an *N*_Am_/*N*_SA_ ratio of 0.38 is explained in more detail as an example.

**Figure 1:**
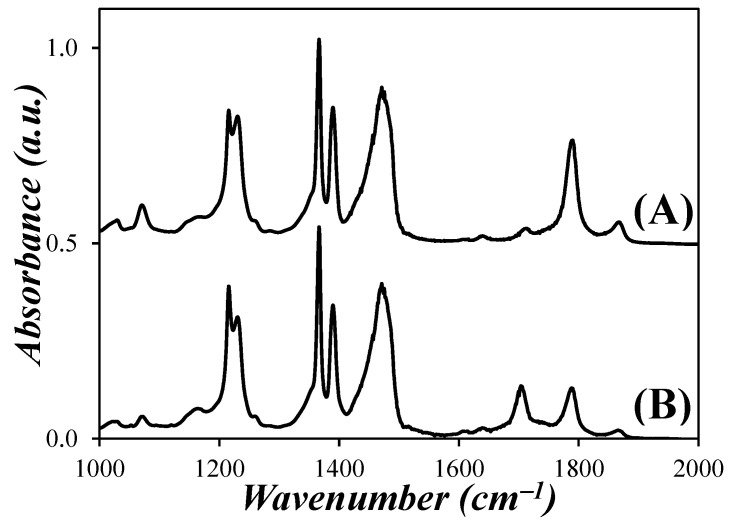
FTIR spectra of precipitated PIBSA: (**A**) Dehydrated and (**B**) after HMDA coupling using an *N*_Am_/*N*_SA_ ratio of 0.38.

PIBSA (6.3 g) was dissolved in 8 mL of THF and precipitated with 200 mL acetone to remove low molecular weight impurities along with the low molecular weight fraction of the MWD. The mixture was then decanted, and PIBSA was dried overnight under vacuum at 70 °C. PIBSA (0.93 g) was dissolved in 20 mL of hot xylene, and the hot PIBSA solution was added to a 100 mL round bottom flask (RBF), which was connected to a Dean-Stark condenser and kept under nitrogen. The solution was refluxed overnight in an oil bath at 175 °C to dehydrate the partially hydrated PIBSA sample and regenerate succinic anhydride from the succinic acid groups. An aliquot was taken the next day to acquire an FTIR spectrum (see Spectrum A in Figure 1) to confirm dehydration of PIBSA via the disappearance of the absorbance peak at 1710 cm^−1^, characteristic of succinic acid carbonyl.

The succinic anhydride content (*λ*_SA_) of 0.418 mmol/g for the PIBSA sample was determined from a combination of pyrene excimer fluorescence, ^1^H NMR, and UV-Vis spectroscopy, as described earlier [19]. HMDA (8.7 mg, 0.075 mmol) was dissolved in 20 mL of hot xylene and added to the reaction vessel containing the dehydrated PIBSA sample (0.39 mmol SA) in xylene to react overnight at reflux. The next morning, an aliquot was taken from the reaction mixture to acquire an FTIR spectrum, shown as Spectrum B in Figure 1. The FTIR spectrum confirmed the completion of the reaction with the presence of the succinimide absorption band at 1705 cm^−1^. The spectrum also lacked strong amide absorption at 1650 cm^−1^, which would have indicated incomplete cyclization of the succinamic acids into succinimides, as observed in the labeling of PIBSA with 1-pyrenemethylamine in previous work [19].

Since the *N*_Am_/*N*_SA_ ratio was below 1, the final product would include both succinimide and succinic anhydride groups, as not enough amines could react with the succinic anhydride groups, resulting in the succinimide and succinic anhydride absorption bands in Spectrum B in Figure 1. After the reaction was complete, the reaction mixture was condensed and dissolved in THF before being transferred to a 20 mL vial. The THF was evaporated under a flow of nitrogen before the polymer product was dried to completion in a vacuum oven at 70 °C overnight to yield the PIBSA–H(0.38) reaction mixture. After being removed from the vacuum oven, the PIBSA–H(0.38) sample was dissolved in THF for GPC analysis. PIBSA–H(*X*) samples were prepared for *N*_Am_/*N*_SA_ ratios ranging from *X* = 0.19 to 1.51 in 0.19 increments, and the different samples were characterized by FTIR and GPC analyses in the same manner.

## 3. Results and Discussion

As illustrated in an earlier publication [35], the reaction of PIBSA with HMDA generates *b*-PIBSI and higher-order PIBSI products, which shift the MWD of the reaction mixture to higher molecular weights. As a result, the GPC traces acquired in that study showed a shift to lower elution volumes. This observation was taken advantage of in the present study to conduct a more detailed analysis of the GPC traces based on sums of Gaussians to determine the weight fraction (*w_PLM_*) of PIBSA-like molecules (PLM), being either a PIBSA reactant, a *m*-PIBSI product, or an unmaleated PIB chain, present in a PIBSA–H(*X*) reaction mixture. The chemical composition of the PIBSA–H(*X*) reaction mixtures was first characterized in terms of their content of SA and succinimide functionalities, which were determined from the analysis of their FTIR spectra.

### 3.1. Analysis of the Fourier-Transform Infrared (FTIR) Spectra

FTIR spectroscopy was used to monitor the coupling of PIBSA with HMDA at different *N*_Am_/*N*_SA_ ratios. The content of SA and succinimide groups was monitored by the intensity of their absorption bands at 1785 and 1705 cm^−1^, respectively [19,33,34]. The FTIR spectra of the PIBSA–H(*X*) coupling products, where *X* is the *N*_Am_/*N*_SA_ ratio used in a given coupling reaction, are shown in Figure S1 in the Supplementary Material. The FTIR spectra in Figure S1 shared most of the same features, notably the characteristic PIB peaks from the methyl swinging motions at 1365 and 1390 cm^−1^ and methylene bending and scissoring peaks at 1225 and 1465 cm^−1^, respectively [19,33,36]. The PIBSA–H(1.51) sample had a small amide peak at 1650 cm^−1^, which was not as prevalent in the other samples with lower *N*_Am_/*N*_SA_ ratios, possibly due to its increased amine content.

**Figure 2:**
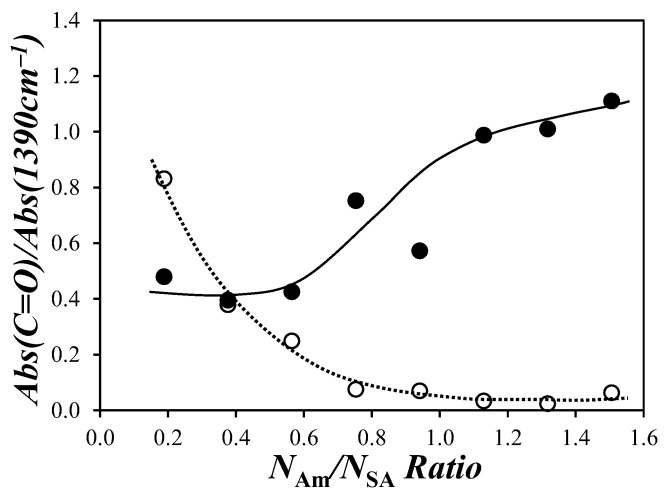
Plot of the (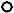) *Abs*(1785 cm^−^^1^)/*Abs*(1390 cm^−^^1^) and (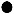) *Abs*(1705 cm^−^^1^)/*Abs*(1390 cm^−^^1^) ratios as a function of the *N*_Am_/*N*_SA_ ratio for the PIBSA–H(*X*) reaction products.

The *Abs*(1785 cm^−^^1^)/*Abs*(1390 cm^−^^1^) and *Abs*(1705 cm^−^^1^)/*Abs*(1390 cm^−^^1^) ratios, taken as measures of the SA and succinimide contents in the reaction mixture, respectively, were plotted as a function of the *N*_Am_/*N*_SA_ ratio in Figure 2 to monitor the composition of the reaction mixture as more hexamethylene diamine was added to the PIBSA sample. The *Abs*(1785 cm^−^^1^)/*Abs*(1390 cm^−^^1^) ratio decreased with increasing *N*_Am_/*N*_SA_ ratio, as expected, approaching zero for an *N*_Am_/*N*_SA_ ratio equal to 1 and remaining close to zero for *N*_Am_/*N*_SA_ ratios greater than 1. The non-zero values of the *Abs*(1785 cm^−^^1^)/*Abs*(1390 cm^−^^1^) ratio for *N*_Am_/*N*_SA_ ratios greater than 1 could be due to the weak secondary absorption band of the succinimide at 1775 cm^−^^1^, which increased in intensity with increasing *N*_Am_/*N*_SA_ ratios (see Figure S1). The *Abs*(1705 cm^−^^1^)/*Abs*(1390 cm^−^^1^) ratio was weak for the PIBSA–H(0.19) product as little succinimide was formed, and it increased with an increasing *N*_Am_/*N*_SA_ ratio, as expected. In summary, the FTIR spectra showed that the coupling reactions between PIBSA and HMDA were efficient and that the IR absorbance of the succinimide and succinic anhydride carbonyls followed the expected trends.

### 3.2. Gel Permeation Chromatography (GPC) Analysis

**Figure 3:**
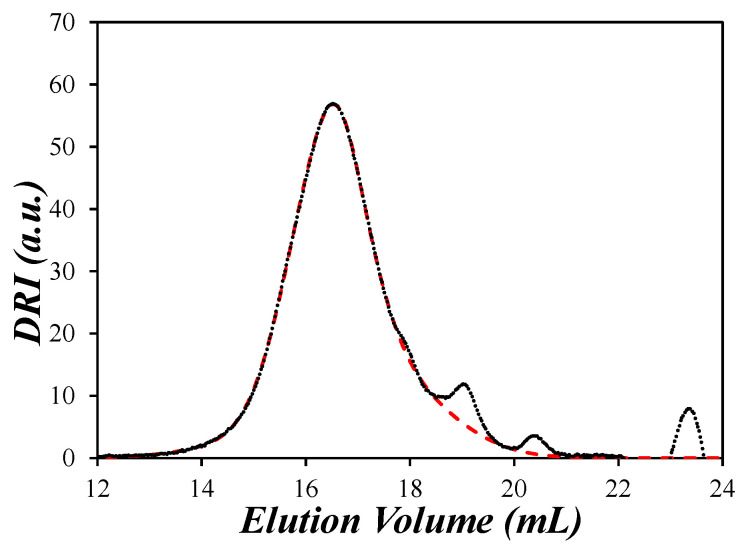
Plot of the (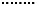) DRI trace and (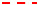) sum of Gaussians fit of a dehydrated PIBSA sample as a function of elution volume. The contributions of the solvent peaks at 19, 20.5, and 23.5 mL were eliminated in the fitted GPC trace.

**Figure 4:**
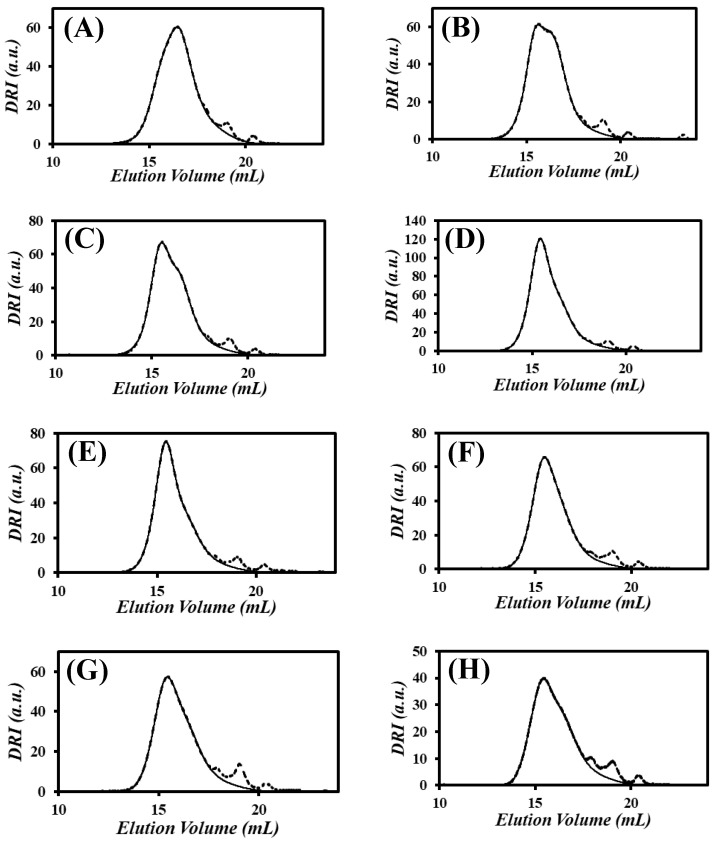
Experimental DRI traces (- - -) from GPC analysis of the PIBSA–H(*X*) products and their fit with a sum of Gaussians (──) obtained for *N*_Am_/*N*_SA_ ratios of (**A**) 0.19, (**B**) 0.38, (**C**) 0.56, (**D**) 0.75, (**E**) 0.94, (**F**) 1.13, (**G**) 1.32, and (**H**) 1.51.

The program *tetra*gauss was used to fit the GPC trace of the PIBSA sample with a sum of four Gaussians, according to Equation (1), and the parameters used in the fit were fixed in the analysis of the GPC traces obtained for the PIBSA–H(*X*) mixtures with the program *x*gaussSNP, according to Equation (2). The *x*gaussSNP programs enabled the isolation of the PLM contribution in the PIBSA–H(*X*) mixtures. The fits of the GPC traces started with one Gaussian whose parameters were allowed to float, adding one Gaussian with floating parameters at a time to the sum, and comparing the squared difference between the experimental and calculated GPC profiles for each fit until the difference showed little variation with an increasing number of Gaussians. This procedure resulted in good fits for the GPC traces, as illustrated in Figure 3 for the PIBSA sample and Figure 4 for all of the PIBSA–H(*X*) reaction mixtures.

The DRI trace in Figure 4A reached its maximum at an elution volume of 16.7 mL, similar to the DRI trace of PIBSA in Figure 3. However, as more HMDA was added to the reaction mixtures, the peak maximum of the DRI traces in Figure 4B–E shifted to a lower elution volume of 15.6 mL while retaining a shoulder on the low molecular weight side corresponding to the contribution from PLM, which decreased with an increasing *N*_Am_/*N*_SA_ ratio. As the *N*_Am_/*N*_SA_ ratio increased past unity, the maximum of the DRI traces in Figure 4F–H remained at 15.6 mL, but the contribution from PLM increased, appearing as a shoulder on the low molecular weight side of the distribution. Finally, one advantage of the fits with sums of Gaussians was the elimination of the small peaks, which appeared at the low molecular weight end of the GPC traces in Figure 3 and Figure 4 and could have been due to residual traces of solvent and/or HMDA remaining in the samples.

**Figure 5:**
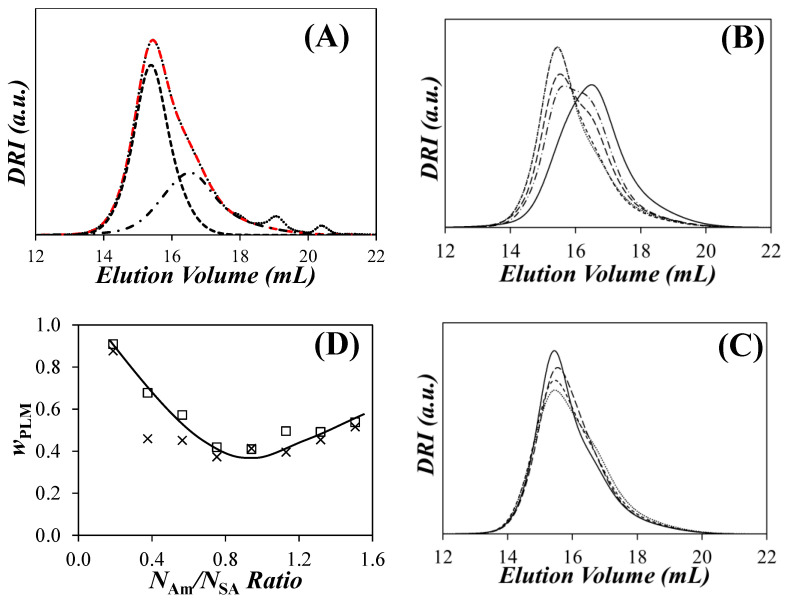
(**A**) An example of the quality of (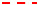) the fit with a sum of Gaussians for (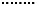) the DRI trace of PIBSA–H(0.75), (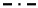) the contribution from PIBSA-like molecules, and (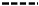) the contribution from non-PIBSA-like molecules. (**B**) Fits with sums of Gaussians for the PIBSA–H(*X*) samples with *N*_Am_/*N*_SA_ ratios of (**B**) (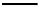) 0.19, (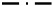) 0.38, (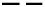) 0.56, (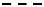) 0.75, and (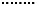) 0.94, and (**C**) (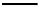) 0.94, (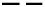) 1.13, (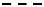) 1.32, and (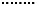) 1.51, normalized to a sum of 1. (**D**) Plots of the weight fraction (*w_PLM_*) of PIBSA-like molecules in the PIBSA–H(*X*) samples determined by fitting the DRI traces with (×) *x*gaussSNP and (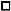) linear regression.

Since the fits of the DRI traces with a sum of Gaussians in Figure 4 gave a good representation of the PIBSA/PIBSI species without the solvent peaks in the PIBSA–H(*X*) mixtures, they were used to represent the experimentally obtained DRI traces and are referred to as the experimental* DRI traces from this point on. Examples of the traces obtained by fitting the PIBSA–H(0.75) DRI trace with *tri*gaussSNP and the comparison of the fits with sums of Gaussians of the PIBSA–H(*X*) samples having *N*_Am_/*N*_SA_ ratios ranging from 0.19 to 0.94 and from 0.94 to 1.51 are shown in Figure 5A–C, respectively.

In the *tri*gaussSNP analyses, PIBSA and *m*-PIBSI were assumed to share the same MWD, and both molecules are referred to as PIBSA-like molecules (PLM). The ability to decompose the MWD of the reaction products into PLM and non-PIBSA-like molecules (*non*-PLM) through the analysis of the DRI traces with sums of Gaussians enabled the determination of the weight fraction (*w_PLM_*) of PLM in the reaction mixture from the ratio *A*/(*A* + *B*), where *A* and *B* are the pre-factors used in Equation (2), since the DRI signal is proportional to the mass concentration of the polymer passing through the detector. A plot of *w_PLM_* as a function of the *N*_Am_/*N*_SA_ ratio is presented in Figure 5D. For *N*_Am_/*N*_SA_ ratios lower than unity, *w_PLM_* first decreased with increasing *N*_Am_/*N*_SA_, as *b*-PIBSI and higher-order PIBSI oligomers were produced in the reaction, until the *N*_Am_/*N*_SA_ ratio reached unity and *w_PLM_* passed through a minimum. As the *N*_Am_/*N*_SA_ ratio increased past unity, more *m*-PIBSI was generated, and *w_PLM_* increased slightly with the increasing *N*_Am_/*N*_SA_ ratio. The most noticeable feature of Figure 5D was that the *w_PLM_* did not reach zero at a *N*_Am_/*N*_SA_ ratio of unity. This observation suggested that a significant number of PIBSA molecules, probably unmaleated PIB chains, did not react with HMDA. Taking the lowest *w_PLM_* value obtained in Figure 5D as the weight fraction of unmaleated PIB (*w_PIB_*) would suggest that 37 wt% of unmaleated PIB chains are present in the PIBSA sample.

**Figure 6:**
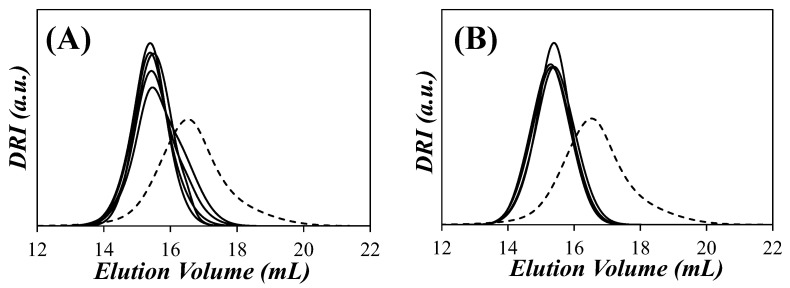
Overlaid DRI contribution of the non-PIBSA fractions of the PIBSA–H(*X*) samples with *N*_Am_/*N*_SA_ ratios of (**A**) 0.19, 0.38, 0.56, 0.75, and 0.94, and (**B**) 0.94, 1.13, 1.32, and 1.51, normalized to a sum of 1. Dashed line: DRI traces of PIBSA.

Comparison of the traces in Figure 5B,C revealed some interesting trends for the PIBSA–H(*X*) products. The MWD shifted to lower elution volumes when the *N*_Am_/*N*_SA_ ratio increased from 0.19 to 0.94 in Figure 5B, as would be expected if more diamines were added to favor the coupling with PIBSA molecules into *b*-PIBSI and higher-order PIBSI products. The high molecular weight region of the DRI traces in Figure 5C appeared to have roughly the same front in the low *V_el_* region and peak maximum, inferring that the same higher-order PIBSI products were generated at *N*_Am_/*N*_SA_ ratios ≥ 1. To further examine this conclusion, the contribution of the *non*-PLM in the experimental* DRI traces was overlaid in Figure 6A,B for *N*_Am_/*N*_SA_ ratios between 0.19 and 0.94 and between 0.94 and 1.51, respectively.

The traces in Figure 6B showed that the *non*-PLM in the PIBSA–H(*X*) mixtures had a similar MWD for all *N*_Am_/*N*_SA_ ratios ≥ 0.94, inferring that the coupling reaction must be producing the same higher-order PIBSI products regardless of the *N*_Am_/*N*_SA_ ratio. The *non*-PLM contribution in the DRI traces for the *N*_Am_/*N*_SA_ ratios of 0.38 and 0.56 shown in Figure 6A had a shoulder in the low MW region of the MWDs, where PIBSA is expected to elute. This observation would imply that the fraction of the PLM in these samples was underestimated, even more so for the PIBSA–H(0.38) sample, which had a larger shoulder.

The main conclusion from the analysis of the DRI traces in Figure 6 is that they could be decomposed into just two contributions: one contribution for PLMs, referring to PIBSA, *m*-PIBSI, or non-maleated PIB chains, which would be indistinguishable; and another contribution for the same higher-order PIBSI product. Since the shape of the DRI traces in Figure 6, which describes the MWD of the higher-order PIBSI products, did not seem to change much with the *N*_Am_/*N*_SA_ ratio, it suggests that the same higher-order PIBSI product was being generated and that this product was *b*-PIBSI. To elaborate further on this proposal, the experimental* DRI traces were fitted with a linear regression, assuming that they were the sum of two contributions, one for PLMs and the other for the *non*-PLM portion of the PIBSA–H(0.94) sample in Figure 6, taken as a representative MWD of the *non*-PLM produced in the PIBSA–H(*X*) reactions. The *w_PLM_* fractions obtained by fitting the experimental* DRI traces of the PIBSA–H(*X*) mixtures with a sum of Gaussian fit or a linear regression were compared in Figure 5D. When plotted against *N*_Am_/*N*_SA_ in Figure 5D, similar *w_PLM_* values were obtained for the PIBSA–H(*X*) reaction mixtures, regardless of the fitting method.

**Figure 7:**
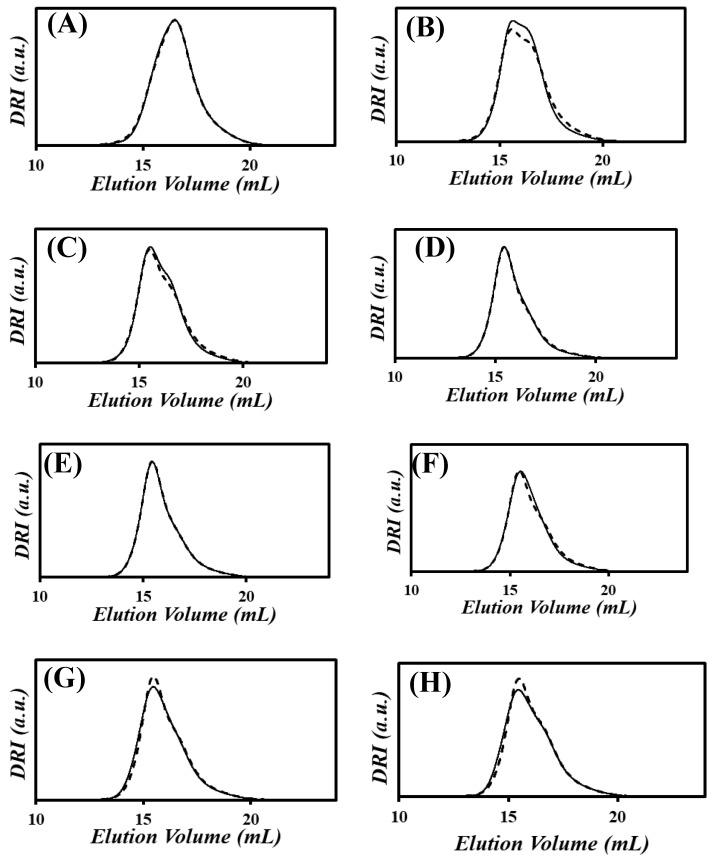
Comparison of the fits obtained with (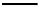) the *x*gaussSNP programs and (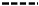) the linear regression analysis of the PIBSA–H(*X*) samples with *N*_Am_/*N*_SA_ ratios of (**A**) 0.19, (**B**) 0.38, (**C**) 0.56, (**D**) 0.75, (**E**) 0.94, (**F**) 1.13, (**G**) 1.32, and (**H**) 1.51.

The DRI traces obtained by applying a linear regression with one contribution for PLMs and another for *non*-PLMs were compared to the experimental* DRI traces in Figure 7. While there were some slight deviations from the experimental* DRI traces near the peak maxima, as in Figure 7B,H, the regressions gave a fairly good representation of the experimental* DRI traces of each PIBSA–H(*X*) sample. The good agreement between the experimental* DRI traces and those synthesized using two set contributions supports the earlier suggestion that the coupling reaction between PIBSA and HMDA might be producing the same higher-order *b*-PIBSI product regardless of the *N*_Am_/*N*_SA_ ratio applied. The good agreement between the results obtained from the fit of the DRI traces with a linear regression or the *x*gaussSNP programs suggests that the proposed analysis could successfully characterize the products of the PIBSA–H(*X*) reactions based only on a GPC instrument with a DRI detector and the Gaussian fitting programs developed in house.

Unfortunately, the experimental validation of the trivial proposal that the MWD shown in Figure 7 can be decomposed into two contributions of PLM and *non*-PLM is challenging for the following reasons. First, three different polymer types, namely PIB, PIBSA composed of PIBSA_1_ and PIBSA_2_, and *m*PIBSI, constitute the PLM and cannot be isolated or individually characterized due to their similar chemical compositions. Second, the MWD of the *b*-PIBSI and higher-order PIBSI species constituting the *non*-PLM shows a significant overlap with the MWD of the PLM in Figure 6, indicating that both PLM and *non*-PLM elute together in the GPC instrument. Third, a complete compositional analysis of all the different species present in a PIBSA–H(*X*) reaction mixture separated according to their size by GPC cannot be accomplished because GPC does not have the resolution capability to distinguish at each elution volume between so many different polymeric species that share a similar chemical composition consisting mostly of PIB and differentiated by only one or two SA or SI groups.

Instead, simulation programs were implemented to predict the molar fractions of different reaction products that could be made in a given PIBSA–H(*X*) reaction. In turn, these molar fractions could be used in combination with the DRI traces corresponding to the PLM and *non*-PLM species in Figure 6 to generate theoretical DRI traces that could be compared with the experimental* DRI traces shown in Figure 7. A reasonable agreement between the simulated and experimental* DRI traces would then be taken as validation of the composition of the different components present in a given PIBSA–H(*X*) reaction mixture that would have been predicted by the simulations. The implementation of these simulations is described in the following section.

### 3.3. Simulations to Predict the Composition of a PIBSA–H(X) Reaction Mixture

To predict the composition of the PIBSA–H(*X*) mixtures in terms of PLM and *non*-PLM contributions, the coupling programs *reaction3* and *reaction4* were created to simulate the products of a coupling reaction between PIBSA and HMDA based on the known molar fraction (*f*_doubly_) of doubly modified PIBSA_2_ in the PIBSA sample and the *N*_Am_/*N*_SA_ ratio used in the reaction. Since the *non*-PLM fraction of the PIBSA–H(*X*) mixtures shared a common MWD, in Figure 6, the proposal was made that the PIBSA–H(*X*) coupling reactions might yield the same higher-order PIBSI product regardless of the *N*_Am_/*N*_SA_ ratio. The similarity of the *non*-PLM products suggested that only *b*-PIBSI dimers were formed, possibly due to the short span of HMDA giving rise to steric hindrance when trying to couple two PIBSAs to the same PIBSA_2_ molecule. If steric hindrance were preventing the formation of *tri*-PIBSA or higher-order PIBSI products in the PIBSA–H(*X*) coupling reactions, only *b*-PIBSI dimers would form, which would result in the same *non*-PLM fraction in all the DRI profiles regardless of the *N*_Am_/*N*_SA_ ratio, as concluded from the GPC analysis shown in Figure 6B. While this would eliminate the formation of higher-order structures such as *tri*-PIBSI, the *f*_doubly_ value for the PIBSA sample of interest would still need to be taken into consideration in simulations to account for the extra succinic anhydride in PIBSA_2_, which would consume the amines of HMDA, but not append an additional PIBSA if the neighboring succinic group of a PIBSA_2_ molecule had already been coupled to another PIBSA to form *b*-PIBSI.

To further study these possibilities, two simulation programs were implemented. The program *reaction3* was created to predict the molar fractions of *m*-PIBSI, *b*-PIBSI, and higher-order PIBSIs expected if the coupling between PIBSA and HMDA was not sterically hindered. Similarly, the *reaction4* program was created to predict the molar fractions of PLMs and *b*-PIBSI products expected from the coupling of PIBSA and HMDA, while assuming that a PIBSA_2_ molecule could react with two diamines but only one other PIBSA molecule due to steric hindrance, which would limit the PIBSI products to only *m*-PIBSI and *b*-PIBSI.

A *f*_doubly_ value of 0.19, determined earlier for the PIBSA sample [19], was used in the *reaction3* and *reaction4* programs. Each of the *reaction3* and *reaction4* programs was carried out with 1000 PIBSA molecules and averaged over 100 simulations using *N*_Am_/*N*_SA_ ratios of 0.19, 0.38, 0.56, 0.75, 0.94, 1.13, 1.32, 1.51, 1.75, and 2.00. For each coupling reaction, the number of product molecules with different numbers of PIB segments (*n*_PIB_) and SA (*n*_SA_) and Am (*n*_Am_) groups was compiled, and the resulting numbers of PIBSA and PIBSI species are listed in Tables S5 and S6 in the Supplementary Material. The numbers of PIBSA and PIBSI molecules were used to calculate the molar fractions *f*_PIBSA_, *f*_PIB1SI_, *f*_PIB2SI_, *f*_PIB3SI_… of the different species PIBSA, *m*-PIBSI, *b*-PIBSI, *tri*-PIBSI…, and these molar fractions were then employed to calculate $w_{PLM}^{sim}$, as shown in Equation (4).

(4) $$w_{PLM}^{sim}\;=\;\frac{f_{\mathrm{PIBSA}}+f_{\mathrm{PIB}1\mathrm{SI}}}{f_{\mathrm{PIBSA}}+f_{\mathrm{PIB}1\mathrm{SI}}+\;\sum_{i=2}^{n}i\times f_{\mathrm{PIB}i\mathrm{SI}}}$$

**Figure 8:**
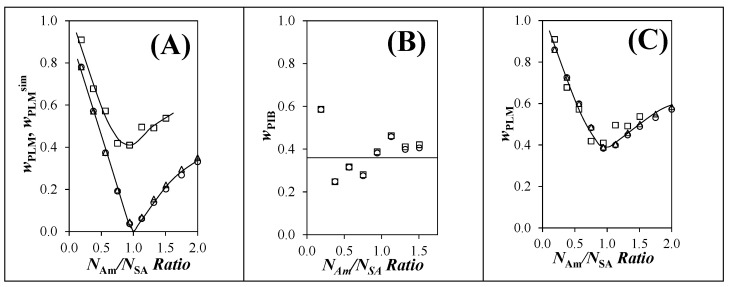
Weight fraction of (**A**) PLM obtained (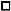) experimentally from regression of GPC traces (Figure 5D) and with (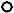) *reaction3* and (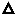) *reaction4*, (**B**) unmaleated PIB obtained with (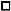) *reaction3* and (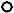) *reaction4*, and (**C**) PLM obtained (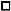) experimentally from regression (Figure 5D) and with (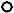) *reaction3* and (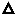) *reaction4* after adding a constant *w_PIB_* of 0.36.

The $w_{PLM}^{sim}$ fractions predicted from both simulation programs were plotted as a function of the *N*_Am_/*N*_SA_ ratio in Figure 8A. $w_{PLM}^{sim}$ decreased steeply as the *N*_Am_/*N*_SA_ ratio increased from 0.19 to 0.94, before increasing gently with increasing *N*_Am_/*N*_SA_ ratios from 0.94 to 2.0. Both simulations yielded similar $w_{PLM}^{sim}$ fractions for all *N*_Am_/*N*_SA_ ratios. The V-shaped $w_{PLM}^{sim}$-*vs*-*N*_Am_/*N*_SA_ plot obtained with the simulations closely mirrored the experimental *w_PLM_* obtained by GPC analysis with sums of Gaussians or linear regression in Figure 5D. The only noticeable difference was that the plot obtained by simulations yielded a zero-$w_{PLM}^{sim}$ fraction for the PIBSA–H(1) reaction mixture, whereas the experimental *w_PLM_* fraction was significantly larger than zero. The only possible explanation for this discrepancy was that the PIBSA sample contained unmaleated PIB chains that did not participate in the coupling reaction with HDMA. To account for the presence of unmaleated PIB in the PIBSA sample, the weight fraction of unmaleated PIB (*w_PIB_*) could be simply estimated from Equation (5), whose derivation is provided in the Supplementary Material.

(5) $$w_{PIB}\;=\;\frac{w_{PLM}-w_{PLM}^{sim}}{1-\;w_{PLM}^{sim}}$$

A plot of *w_PIB_* as a function of *N*_Am_/*N*_SA_ is shown in Figure 8B. Excepting the *w_PIB_* value obtained for an *N*_Am_/*N*_SA_ ratio of 0.19, which was probably an outlier, the *w_PIB_* values shown in Figure 8B remained fairly constant with the *N*_Am_/*N*_SA_ ratio and took an average *w_PIB_* value of 0.36 (±0.08) when calculated with either *reaction3* or *reaction4*. This analysis suggested that 36 wt% of the PIBSA sample was unmaleated PIB, in good agreement with the 37 wt% prediction, which was made earlier based on the trends shown in Figure 5D. The same weight fraction *w_PIB_* of 0.36 was then used to calculate $w_{PLM}^{cal}$ equal to $w_{PLM}^{sim}$ + *w_PIB_* × (1 − $w_{PLM}^{sim}$) based on Equation (5), which was compared in Figure 8C to the experimental *w_PLM_* fractions obtained from the regression fits in Figure 5A for the PIBSA–H(*X*) mixtures. Without including *w_PIB_*, $w_{PLM}^{sim}$ showed a similar trend in Figure 8A to that of *w_PLM_* obtained experimentally by GPC analysis, but $w_{PLM}^{sim}$ was shifted to lower values. Upon including *w_PIB_* equal to 0.36 in the calculation of $w_{PLM}^{cal}$, good agreement was obtained between the calculated $w_{PLM}^{cal}$ and experimental *w_PLM_* weight fractions in Figure 8C.

### 3.4. Predicted MWD of b-PIBSI Supports That b-PIBSI Is the Only Higher-Order PIBSI Product

**Figure 9:**
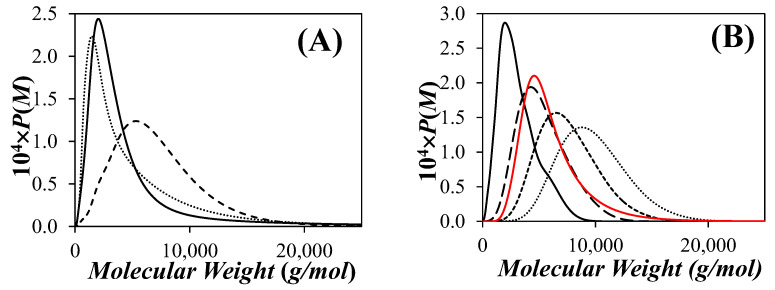
Plots of *P*(*M*) as a function of molecular weight for (**A**) the PIB standard with *M*_n_ equal to (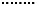) 1.0, (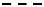) 3.2 kg/mol, and (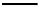) PIBSA and (**B**) simulated MWD for (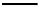, *P*_1_(*M*)) *m*-PIBSI, (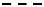, *P*_2_(*M*)) *b*-PIBSI, (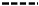, *P*_3_(*M*)) *tri*-PIBSI, (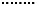, *P*_4_(*M*)) *tetra*-PIBSI, and (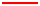) experimental MWD for higher-order products in the PIBSA–H(0.94) reaction mixture.

A procedure was implemented as in the Supplementary Material to establish a calibration curve (see Equation (S13)) for the GPC instrument based on PIB standards having a broad MWD. The calibration curve was applied to the DRI trace obtained for the PIBSA sample to obtain its MWD, given by *P*(*M*) in Equation (3), also referred to as *P*_1_(*M*) for the PIBSA unimer. *P*_1_(*M*) was plotted as a function of *M* in Figure 9A. Equations (S10) and (S11) could be applied to determine the number (*M*_n_) and weight (*M*_w_) average molecular weight of PIBSA, which were found to equal 1.3 and 2.6 kg/mol, respectively. The *M*_n_ value of 1.3 kg/mol agreed with the location of the DRI trace of PIBSA with respect to the two PIB standards in Figure 9A, since it was much closer to the DRI trace of the PIB standard with an *M*_n_ equal to 1.0 kg/mol compared to that of the PIB standard with an *M*_n_ of 3.2 kg/mol.

*P*_1_(*M*), shown as a solid trace in Figure 9A, was used to predict the MWD of higher-order PIBSI oligomers using the *prod-dis2* coupling program. The program worked by transforming *P*_1_(*M*) into *P*_1_(*i*), where *i* is the degree of polymerization of a PIBSA molecule having a molecular weight *M* (*i* = *M*/*M*_o_, where *M*_o_ is the molar mass of an isobutylene structural unit equal to 56.11 g/mol). The probability *P*_z_(*i*) of a higher-order (z = 2, 3,…) PIBSI product could then be obtained with Equation (6), where the probability *P*_z−1_(*i*) represents the MWD of the z − 1 order PIBSI product. For example, coupling the MWD (*P*_1_(*i*)) of PIBSA with itself yields the MWD (*P*_2_(*i*)) of *b*-PIBSI, coupling the MWDs of (*P*_1_(*i*)) PIBSA and (*P*_2_(*i*)) *b*-PIBSI yields the MWD (*P*_3_(*i*)) of *tri*-PIBSI, etc.

(6) $$P_{z}(i)\;=\;\sum_{k=1}^{i}P_{1}(k)\times P_{z-1}(i+1-k)$$

Equation (6) was applied to determine all of the functions *P*_z_(*M*) required for the higher-order PIBSI constructs obtained in the simulations, but only the functions *P*_1_(*M*), *P*_2_(*M*), *P*_3_(*M*), and *P*_4_(*M*) were plotted as a function of *M* in Figure 9B. The functions shifted to higher molecular weights with increasing *z*, as would be expected since a higher *z* indicates a higher order and thus a larger PIBSI product. However, the increase in molecular weight should be considered an apparent increase since PIBSI products of orders higher than 2 have a structure closer to that of a star polymer with a hydrodynamic volume (*V*_h_) that is smaller than that of the linear polymer of an equivalent molecular weight. Since the calibration curve of the GPC instrument is based on linear PIB standards, the molecular weights predicted with Equation (6), which assumes that the coupling between linear PIB molecules results in longer linear PIB chains, would predict larger molecular weights than those found from the GPC analysis based on the calibration curve given in Equation (S13). Consequently, the MWD predicted with Equation (6) for *b*-PIBSI should be correct since it is a linear polymer, but that for *tri*-PIBSI and higher-order PIBSI products should be overestimated. Nevertheless, these latter PIBSI products should yield an MWD that is shifted to higher molecular weights compared to that of *b*-PIBSI, albeit with a smaller shift than predicted in Figure 9B.

In order to assess how the MWD for the higher PIBSI products generated in the PIBSA–H(*X*) reactions found experimentally in Figure 6B would compare to those predicted from Equation (6), the MWD of the higher-order PIBSI products obtained for the PIBSA–H(0.94) reaction in Figure 6B was compared to the *P*_z_(*M*) distributions with *z* equal to 1–4 in Figure 9B. The MWD obtained for the higher-order products in the PIBSA–H(0.94) reaction mixture had been found to be representative of the higher PIBSI products generated in all of the PIBSA–H(*X*) reactions with *X* ≥ 0.94 (see Figure 6B). As it turns out, the MWD of the higher-order products found for the PIBSA–H(0.94) reaction appeared to match remarkably well the *P*_2_(*M*) function in Figure 9B, further supporting the notion that *b*-PIBSI was the highest-order product generated in the PIBSA–H(*X*) reactions.

**Figure 10:**
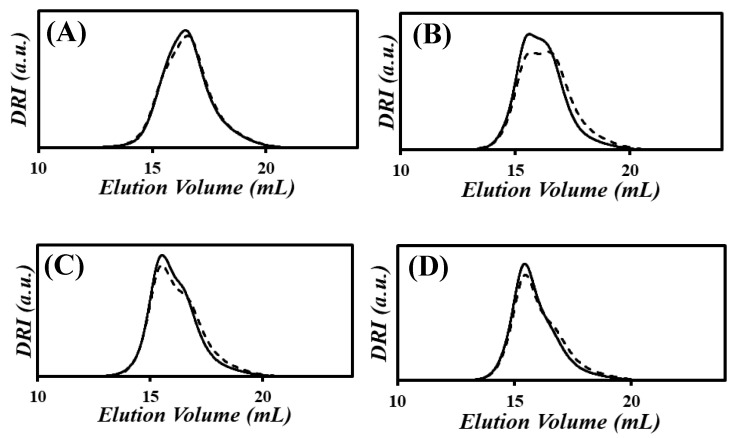

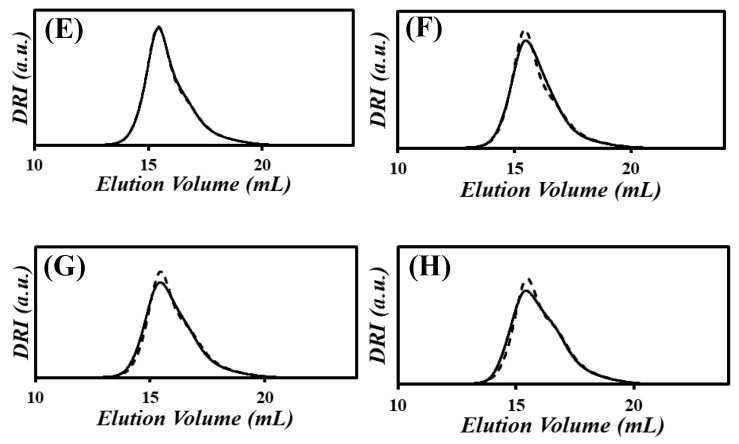
Comparison of (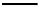) the simulated DRI traces obtained from the molar fractions obtained with *reaction4*, *w_PIB_* = 0.36, and the DRI traces of PIBSA and *b*-PIBSI given by the GPC trace obtained for the *non*-PLM of PIBSA–H(0.94) in Figure 6, and (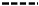) the experimental* DRI traces fitted with the *x*gaussSNP programs of the PIBSA–H(*X*) products. The *N*_Am_/*N*_SA_ ratios equal (**A**) 0.19, (**B**) 0.38, (**C**) 0.56, (**D**) 0.75, (**E**) 0.94, (**F**) 1.13, (**G**) 1.32, and (**H**) 1.51.

Since all lines of investigation support that *b*-PIBSI is the highest-order product generated in the PIBSA–H(*X*) reactions, synthetic DRI traces were built for the PIBSA–H(*X*) reaction mixtures based on the molar fractions *f*_PIB_, *f*_PIBSA_, *f*_PIB1SI_, and *f*_PIB2SI_ obtained in Table S6 with the simulation program *reaction4* and the MWD obtained for PIBSA and the higher-order products in the PIBSA–H(0.94) mixture. The resulting DRI traces were then compared in Figure 10 to the experimental* DRI traces. 

The results shown in Figure 10 indicate strong agreement between the experimental* and synthesized DRI traces. Since the synthesized DRI traces were based on the molar fractions obtained with the simulation program *reaction4*, which assumed that steric hindrance prevented the formation of *tri*-PIBSI and higher-order PIBSI products, the good agreement between the traces shown in Figure 10 further supports this claim. This good agreement also validates the composition of the PIBSA–H(*X*) reaction mixtures determined through the simulation programs, as such detailed information about the different PIB species present in the reaction mixture would be difficult to determine experimentally.

**Figure 11:**
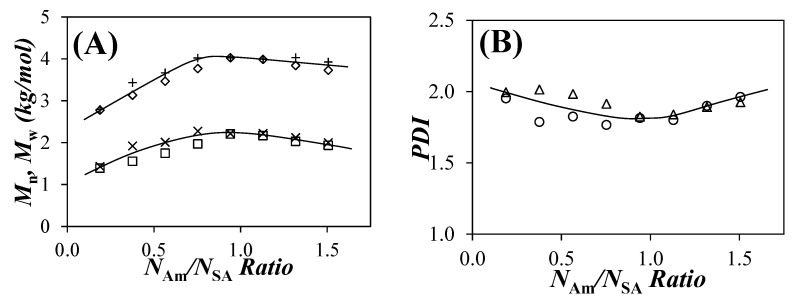
Plot of (**A**) (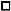,×) *M*_n_ and (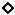,+) *M*_w_ and (**B**) (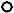,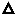) PDI of the PIBSA–H(*X*) samples as a function of the *N*_Am_/*N*_SA_ ratio, (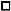,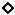,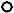) simulations, and (×,+,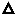) experiments.

The synthesized and experimental* DRI traces shown in Figure 10 were then analyzed using the calibration curve in Equation (S13), which was generated for the GPC instrument, to obtain *M*_n_, *M*_w_, and the polydispersity index (PDI) for the PIBSA–H(*X*) reaction mixtures. They are plotted as a function of the *N*_Am_/*N*_SA_ ratio in Figure 11. The *M*_n_ and *M*_w_ values increased when the *N*_Am_/*N*_SA_ ratio increased from 0.19 to 0.94, before decreasing slightly when the *N*_Am_/*N*_SA_ ratio increased from 0.94 to 1.51. These trends mirrored the large decrease in the experimental weight fraction of PLM (*w_PLM_*) upon increasing the *N*_Am_/*N*_SA_ ratio from 0.19 to 0.94, before showing a slight increase when the *N*_Am_/*N*_SA_ ratio increased further from 0.94 to 1.51, as seen in Figure 8. Both trends reflected the shift towards the formation of more *b*-PIBSI products, as the *N*_Am_/*N*_SA_ ratio increased from 0.19 to 0.94 before more *m*-PIBSI was generated when the *N*_Am_/*N*_SA_ ratio increased from 0.94 to 1.51. In summary, the experimental results seem to agree well with the simulated data, further supporting the conclusion that 36 wt% of unmaleated PIB was present in the PIBSA sample and that steric hindrance prevents the formation of PIBSI products with more than two PIB chains. Taking the *f*_doubly_ and *w_PIB_* values of 0.19 and 0.36, respectively, the PIBSA chains could be determined to be composed of 50, 38, and 12 mol% of singly maleated, unmaleated, and doubly maleated PIB chains, respectively. Consequently, the proposed methodology that was applied to this PIBSA sample provided a complete characterization of the PIBSA chain ends.

## 4. Conclusions

PIBSA molecules are an essential component in the preparation of PIBSI dispersants used as engine oil additives. Since the properties of PIBSI dispersants are a result of their chemical architecture, which is itself a direct consequence of the chemical composition of the PIBSA starting material, the characterization of PIBSA samples has been the focus of intense research [8,23,24,37,38,39,40,41,42]. Despite the numerous challenges complicating the characterization of PIBSA samples [19], this study provides a protocol that can be implemented to determine the weight fraction *w_PIB_* of unmaleated PIB chains in a PIBSA sample by analyzing the GPC traces obtained with a DRI detector of PIBSA–H(*X*) reaction mixtures obtained from the reaction between different amounts of PIBSA and HMDA. Furthermore, a theoretical framework was introduced whereby the composition of PIBSI products could be predicted from simulations based on the knowledge of *w_PIB_*, *f*_doubly_, and *λ*_SA_ used to characterize the PIBSA sample and the ratio of *N*_Am_/*N*_SA_, reflecting the amounts of PIBSA and HMDA used in a PIBSA–H(*X*) reaction.

To the best of our knowledge, this study represents the most extensive investigation reported in the literature aiming to characterize the chemical composition of PIBSA, a key building block in the preparation of PIBSI dispersants used as engine oil additives. It also introduces a general method based on the coupling of two end-modified linear chains that yield the weight fraction of non-modified chains, an important aspect in the characterization of EMPs used in the preparation of large macromolecules with complex architectures [6,7,8,9,10,11,12,13,14,15,16,17].

## Supplementary Materials

The following are available online at https://www.mdpi.com/article/10.3390/polym15102350/s1, Figure S1: FTIR spectra for the PIBSI–H(*X*) samples, Table S1: *M*_n_ and *M*_w_ values of the PIB standards used for the GPC calibration and determined using the calibration curve from the program *cal-2*, Table S2: Pre-Gaussian factors, averages, and standard deviations retrieved from the fit of the DRI trace of the PIBSA sample as a function of (*V**_el_*) weight tetragauss according to Equation (1), Table S3: Pre-Gaussian factors, averages, and standard deviations retrieved from the fit of the DRI traces of the PIBSA–H(*X*) samples as a function of (*V**_el_*) with *x*gaussSNP (*x* = *bi*, *tri*) according to Equation (2), Table S4: Pre-Gaussian factors, averages, and standard deviations retrieved from the fit of the simulated MWDs obtained for *m*-, *b*-, *tri*-, *tetra*-, *penta*-, *hexa*-, *hepta*-, *octa*-, and *nona*-PIBSI as a function of (*X*_n_) with *x*gauss (*x* = *penta,* and *hexa*) according to Equation (1), Table S5: PIBSI products simulated by the *reaction3* coupling program, Table S6: PIBSI products simulated by the *reaction4* coupling program. Methodology for the GPC calibration curve determination; FTIR spectra; Gaussian fitting parameters; PIBSA coupling products retrieved from simulation programs; determination of *w_PIB_*.
