# Supplementary material for: Characterization of Polyisobutylene Succinic Anhydride (PIBSA) and Its PIBSI Products from the Reaction of PIBSA with Hexamethylene Diamine
Source: Polymers (Basel). 2023 May 17;15(10):2350. doi: 10.3390/polym15102350 (PMC10221681; doi:10.3390/polym15102350)
Supplement: Supplementary file 1 [file polymers-15-02350-s001.zip › polymers-2315084-supplementary.pdf]

Supporting Information for

Characterization of Polyisobutylene Succinic Anhydride (PIBSA) and its PIBSI Products from the Reaction of PIBSA with Hexamethylene Diamine

Franklin Frasca and Jean Duhamel*

Institute for Polymer Research, Waterloo Institute for Nanotechnology, Department of Chemistry, University of Waterloo, Waterloo, ON, N2L 3G1, Canada

* To Whom correspondence should be addressed.

E-mail: fofrasca@uwaterloo.ca; jduhamel@uwaterloo.ca

Contents

A) GPC Calibration Curve Determination.. S2

B) FTIR Spectra.. S6

C) Gaussian Fitting Parameters S7

D) PIBSA Coupling Products Retrieved from *reaction3* and *reaction4* S9

E) Determination of w_{PIB} …………………………………………………………………….. S10

A) GPC Calibration Based on Polydisperse PIB Standards

As discussed in the main text, only polydisperse PIB standards such as those listed in Table S1 are currently available in the range of molecular weights covering the molecular weight distribution (MWD) of typical PIBSA sample (1,000 to 2,500 g/mol) and the PIBSI products generated from the reaction of PIBSA with hexamethylene diamine (HMDA). While this makes the calibration of a GPC instrument challenging, the GPC instrument can still be calibrated by applying the following procedure. First, the calibration curve between the logarithm of the polymer molecular weight (*Ln*(*M*)) is assumed to obey a polynomial of degree *n* with elution volume (V_{el}) as described in Equation S1.

$$Ln(M) = \sum_{i=0}^{n} b_i V_{el}^i \tag{S1}$$

Since the signal DRI(V_{el}) from the DRI detector is proportional to the mass of polymer passing through the detector, it is related to the number average molecular weight (M_n) of the polymer as shown in Equation S2, where *m* is the total mass of polymer sample injected into the GPC instrument, *N* is the number of chains in the polymer sample, *P*(*M*) is the probability for the polymer to have a molar mass *M*, and *K* is a scaling constant.

$$M_n = \frac{m}{N} = \int_0^\infty P(M)MdM = K\int_0^\infty DRI(V_{el})dV_{el} \tag{S2}$$

To obtain an expression for *P*(*M*), Equation S2 can be rewritten in terms of Equation S3.

$$P(M)MdM = K \times DRI(V_{el})dV_{el} \tag{S3}$$

Equation S3 can then be re-arranged to find $P(M)$ as shown in Equation S4.

$$P(M) = K \times \frac{DRI(V_{el})}{M} \frac{dV_{el}}{dM} \tag{S4}$$

Assuming that the calibration curve for the GPC instrument can be approximated by the polynomial function shown in Equation S1, the derivative dV_{el}/dM is given by Equation S5.

$$\frac{dV_{el}}{dM} = \frac{1}{M \sum_{i=1}^{n} i b_i V_{el}^{i-1}} \tag{S5}$$

Combining Equations S4 and S5 yields a new expression for $P(M)$ in Equation S6.

$$P(M) = K \frac{DRI(V_{el})}{M^2 \sum_{i=1}^{n} i b_i V_{el}^{i-1}} \tag{S6}$$

The scaling constant K can be found by setting the integral of $P(M)$ to equal unity as shown in Equation S7 and solving for K in Equation S8.

$$1 = \int_0^\infty P(M) dM = K \int_0^\infty \frac{DRI(V_{el})}{M^2 \sum_{i=1}^{n} i b_i V_{el}^{i-1}} \frac{dM}{dV_{el}} dV_{el} = K \int_0^\infty \frac{DRI(V_{el})}{M} dV_{el} = K \int_0^\infty \frac{DRI(V_{el})}{\exp\left(\sum_{i=0}^{n} b_i V_{el}^i \right)} dV_{el} \tag{S7}$$

$$K = \frac{1}{\int_0^\infty DRI(V_{el}) \exp\left(-\sum_{i=0}^{n} b_i V_{el}^i \right) dV_{el}} \tag{S8}$$

Combining Equations S6 and S8 yields the expression of $P(M)$ in Equation S9 as a function of the DRI detector signal and the parameters b_i used for the calibration curve in Equation S1.

$$P(M) = \frac{1}{\int_0^\infty DRI(V_{el}) \exp\left(-\sum_{i=0}^{n} b_i V_{el}^i\right) dV_{el}} \frac{DRI(V_{el})}{\exp\left(2\sum_{i=0}^{n} b_i V_{el}^i\right) \sum_{i=1}^{n} i b_i V_{el}^{i-1}} \qquad \text{(S9)}$$

The GPC instrument was calibrated with the fairly polydisperse PIB standards with j = **1**, **2**, and **3**, which were purchased from Polymer Source and whose number ($M_{j,n}$) and weight ($M_{j,w}$) average molecular weights are listed in Table S1, where the index j indicates one of the three PIB standards listed in Table S1. Assuming that the calibration curve could be approximated by the polynomial of degree n shown in Equation S1, the predicted $M_{j,n}^{theo}$ and $M_{j,w}^{theo}$ were calculated with Equations S10 and S11 based on the expression of $P(M)$ given in Equation S9. Equations S10 and S11 express the molecular weights $M_{j,n}^{theo}$ and $M_{j,w}^{theo}$ as a function of the polynomial constants b_i from Equation S1 and the $DRI_j(V_{el})$ trace showing the DRI signal of the jth PIB standard as a function of elution volume (V_{el}) obtained from GPC. A second order polynomial (n = 2 in Equation S1) was deemed satisfactory for the calibration curve. The program *cal-2* was then used to minimize the χ^2 function in Equation S12 to optimize the pre-factors b_i in Equation S1 with the Marquardt-Levenberg algorithm to obtain the target $M_{j,n}$ and $M_{j,w}$ values of the 3 PIB standards (i.e. m = 3 in Equation S12) based on their $DRI(V_{el})$ traces and Equations S10 and S11.

$$M_{j,n}^{theo} = \int_0^\infty P(M) M dM = \frac{\int_o^\infty DRI_j(V_{el}) dV_{el}}{\int_o^\infty DRI_j(V_{el}) \exp(-\sum_{i=0}^{n} b_i V_{el}^i)\, dV_{el}} \qquad \text{(S10)}$$

$$M_{j,w}^{theo} = \frac{\int_0^\infty P(M) M^2 dM}{\int_0^\infty P(M) M dM} = \frac{\int_0^\infty DRI_j(V_{el}) \exp(\sum_{i=0}^{n} b_i V_{el}^i)\, dV_{el}}{\int_0^\infty DRI_j(V_{el})\, dV_{el}} \qquad \text{(S11)}$$

$$\chi^2 = \sum_{j=1}^{m}\left(M_{j,n}^{theo} - M_{j,n}\right)^2 + \sum_{j=1}^{m}\left(M_{j,w}^{theo} - M_{j,w}\right)^2 \quad \text{(S12)}$$

Table S1. M_n and M_w values of the PIB standards used for the GPC calibration and determined using the calibration curve from the program *cal-2*.

	**Known**		**From calibration curve**	
PIB standards	M_n (kg/mol)	M_w (kg/mol)	M_n^{theo} (kg/mol)	M_w^{theo} (kg/mol)
**1**	1.0	1.5	1.2	2.4
**2**	3.2	5.0	2.9	5.3
**3**	6.0	7.8	5.6	7.7

Unlike the standard GPC calibration method using standards with narrow MWDs, the program *cal-2* takes the baseline-corrected DRI traces of the PIB standards having broad MWDs and optimizes the b_i parameters in Equation S1 to yield predicted M_n^{theo} and M_w^{theo} values for the PIB standards that would be as close as possible to the M_n and M_w values provided by Polymer Source for the PIB standards **1**, **2**, and **3**. The known M_n and M_w values in Table S1 were found to agree fairly well with the M_n^{theo} and M_w^{theo} values predicted from the calibration curve given in Equation S13 and obtained with *cal-2*, albeit with a larger deviation from 1.5 to 2.4 for the M_w values of the PIB standard **1**.

$$Ln(M) = 0.867\times10^{-2}\times V_{el}^2 - 0.742\times V_{el} + 19.638 \quad \text{(S13)}$$

B) FTIR Spectra

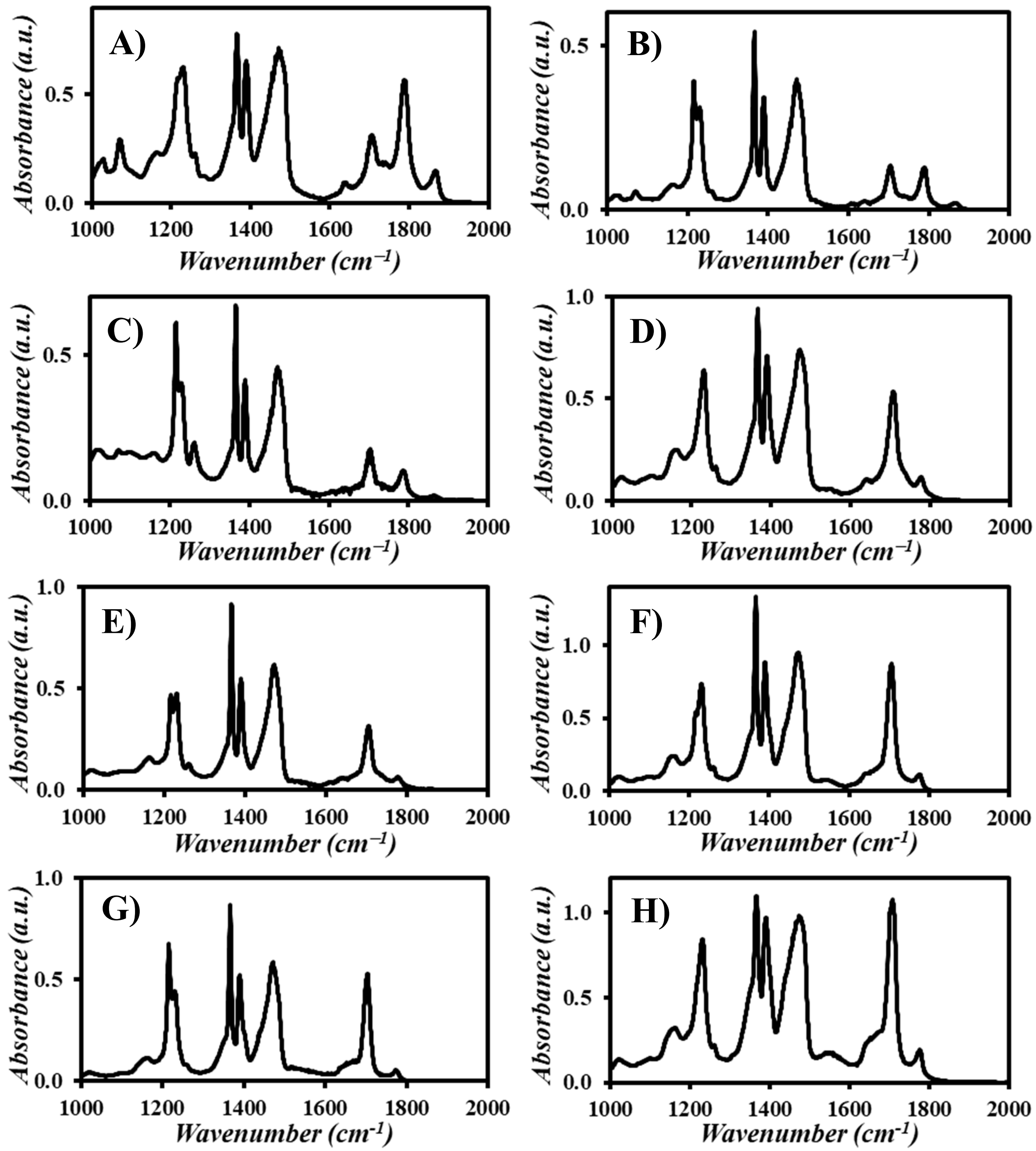

Figure S1. FTIR spectra of the PIBSA-H(*X*) products obtained with N_{Am}/N_{SA} ratios of A) 0.19, B) 0.38, C) 0.56, D) 0.75, E) 0.94, F) 1.13, G) 1.32, and H) 1.51.

C) Gaussian Fitting Parameters

Table S2. Pre-Gaussian factors, averages, and standard deviations retrieved from the fit of the DRI trace of the PIBSA sample as a function of (V_{el}) weight tetragauss according to Equation 1.

Sample	A	a_1	μ_1	σ_1	a_2	μ_2	σ_2	a_3	μ_3	σ_3	a_4	μ_4	σ_4
PIBSA	127.94	0.082	15.563	1.357	0.601	16.399	0.738	0.036	16.661	0.348	0.281	17.505	1.199

Table S3. Pre-Gaussian factors, averages, and standard deviations retrieved from the fit of the DRI traces of the PIBSA-H(X) samples as a function of (V_{el}) with $x$gaussSNP (x = bi, tri) according to Equation 2.

Sample	A	B	a_1	μ_1	σ_1	a_2	μ_2	σ_2	a_3	μ_3	σ_3
PIBSA-H(0.19)	128.933	18.076	0.982	15.494	0.546	0.018	16.497	0.169			
PIBSA-H(0.38)	68.705	80.864	0.120	14.898	0.493	0.170	15.385	0.319	0.710	15.919	0.728
PIBSA-H(0.56)	68.352	82.959	0.307	15.378	0.390	0.679	15.623	0.787	0.014	16.500	0.244
PIBSA-H(0.75)	86.580	145.510	0.465	15.292	0.621	0.225	15.377	0.345	0.310	15.675	0.684
PIBSA-H(0.94)	59.696	84.819	0.351	15.246	0.644	0.186	15.401	0.330	0.463	15.447	0.568
PIBSA-H(1.13)	54.950	83.962	0.029	14.716	0.572	0.051	15.333	0.287	0.920	15.45	0.629
PIBSA-H(1.32)	59.568	71.565	0.189	14.785	0.507	0.070	15.267	0.322	0.741	15.477	0.583
PIBSA-H(1.51)	50.175	47.09	0.230	14.729	0.445	0.096	15.257	0.322	0.674	15.484	0.550

Table S4. Pre-Gaussian factors, averages, and standard deviations retrieved from the fit of the simulated MWDs obtained for *m*-, *b*-, *tri*-, *tetra*-, *penta*-, *hexa*-, *hepta*-, *octa*-, and *nona*-PIBSI as a function of (X_n) with *x*gauss (*x* = *penta,* and *hexa*) according to Equation 1.

Sample	A	a_1	μ_1	σ_1	a_2	μ_2	σ_2	a_3	μ_3	σ_3	a_4	μ_4	σ_4	a_5	μ_5	σ_5	a_6	μ_6	σ_6
m-PIBSI	1	0.035	6.255	2.915	0.075	12.449	4.813	0.389	25.024	9.493	0.279	42.226	11.117	0.099	64.046	12.363	0.123	83.911	27.440
b-PIBSI	1	0.059	33.107	11.459	0.231	50.942	14.965	0.338	71.656	19.543	0.140	128.964	36.421	0.232	98.017	26.432			
tri-PIBSI	1	0.222	147.185	32.072	0.455	105.381	27.707	0.152	81.210	19.496	0.132	174.908	46.604	0.039	59.199	15.010			
tetra-PIBSI	1	0.201	115.857	24.775	0.048	88.669	20.091	0.312	182.506	39.772	0.120	223.575	52.787	0.319	145.913	30.775			
penta-PIBSI	1	0.046	119.008	24.031	0.200	150.470	29.224	0.312	225.445	45.289	0.114	269.378	58.988	0.328	185.351	35.944			
hexa-PIBSI	1	0.044	149.996	27.647	0.195	185.250	33.264	0.314	267.931	50.405	0.109	314.567	64.643	0.338	224.319	40.576			
hepta-PIBSI	1	0.162	218.519	35.602	0.385	300.432	56.306	0.043	180.483	30.633	0.120	354.185	70.166	0.290	258.868	43.395			
octa-PIBSI	1	0.040	213.613	34.232	0.186	255.702	40.571	0.349	301.513	48.721	0.104	402.508	74.672	0.321	351.255	59.420			
nona-PIBSI	1	0.104	258.588	39.993	0.202	304.174	41.717	0.260	349.877	47.994	0.307	392.778	61.581	0.127	437.374	80.016			

D) PIBSA Coupling Products Retrieved from reaction3 and reaction4

Table S5. PIBSI products simulated by the reaction3 coupling program.

N_{Am}/N_{SA} ratio	reaction3.py								
	**PIBSA/ *m*-PIBSI**	***b*- PIBSI**	***tri*- PIBSI**	***tet*- PIBSI**	***penta*- PIBSI**	***hexa*- PIBSI**	***hepta*- PIBSI**	***octa*- PIBSI**	***nona*- PIBSI**
**0.19**	780.22	103.71	3.93	0.13	0.01	0	0	0	0
**0.38**	571.25	187.10	16.14	1.42	0.09	0	0	0	0
**0.56**	373.59	250.26	35.45	4.05	0.57	0.07	0.01	0	0
**0.75**	191.70	288.67	58.49	10.81	1.87	0.36	0.07	0.02	0.01
**0.94**	36.91	290.33	80.39	23.83	6.16	1.78	0.42	0.10	0.08
**1.13**	61.07	282.46	79.45	22.93	5.95	1.61	0.47	0.11	0.04
**1.32**	137.48	278.42	68.80	17.52	4.30	0.99	0.16	0.08	0
**1.51**	201.37	269.81	61.87	13.76	2.87	0.50	0.11	0.03	0
**1.75**	267.90	260.91	52.47	10.23	1.71	0.45	0.10	0	0
**2.00**	330.74	245.20	45.51	8.37	1.32	0.34	0.03	0	0

Table S6. PIBSI products simulated by the reaction4 coupling program.

N_{Am}/N_{SA} ratio	reaction4.py	
	**PIBSA/ *m*-PIBSI**	***b*-PIBSI**
**0.19**	780.78	109.61
**0.38**	570.66	214.67
**0.56**	374.92	312.54
**0.75**	197.30	401.35
**0.94**	45.56	477.22
**1.13**	68.66	465.67
**1.32**	154.44	422.78
**1.51**	221.34	389.33
**1.75**	295.68	352.16
**2.00**	350.26	324.87

E) Determination of w_{PIB}:

The difference between the experimental w_{PLM} and simulated w_{PLM}^{sim} was attributed to the presence of unmaleated PIB in the PIBSA sample, that would not react with HMDA. Using this insight, the experimental and simulation results were combined to estimate the weight fraction w_{PIB} of unmaleated PIB in the PIBSA sample. In Equation S14, f_{PIB}, f_{PIBSA}, and f_{PIBiSI} are the molar fraction of unmaleated PIB, PIBSA, and PIBSI products bearing *i* PIB chains, respectively.

$$w_{PLM} = \frac{f_{PIB} + f_{PIBSA} + f_{PIB1SI}}{f_{PIB} + f_{PIBSA} + f_{PIB1SI} + \sum_{i=2}^{n} i \times f_{PIBiSI}} \tag{S14}$$

Equation S14 can be rearranged to yield the molar fraction f_{PIB} given in Equation S15.

$$f_{PIB} = \frac{(f_{PIBSA} + f_{PIB1SI})(1 - w_{PLM}) - w_{PLM} \sum_{i=2}^{n} i \times f_{PIBiSI}}{w_{PLM} - 1} \tag{S15}$$

The weight fraction w_{PIB} of unmaleated PIB in the PIBSA sample can then be determined by applying Equation S16.

$$w_{PIB} = \frac{f_{PIB}}{f_{PIB} + f_{PIBSA} + f_{PIB1SI} + \sum_{i=2}^{n} i \times f_{PIBiSI}} \tag{S16}$$

Taking advantage of the expression of w_{PIB}^{sim} given as Equation 4 in the main text and using Equation S15 to replace f_{PIB} in Equation S16, an expression for w_{PIB} was obtained in Equation S17.

$$w_{PIB} = \frac{w_{PLM} - w_{PLM}^{sim}}{1 - w_{PLM}^{sim}} \tag{S17}$$
